# A Case of Psoas Abscess, Appearing at the Crural Ring without Any Premonitory Symptoms

**Published:** 1859-07

**Authors:** J. B. Trembley


ART. II.—*A Case of Psoas Abscess, appearing at the Crural Ring without any Premonitory Symptoms.* BY J. B. TREMBLEY, M. D.

In August, 1857, an Englishman, aged 26, came to me laboring under a recent inguinal hernia of the left side, produced by violent exertions in pitching hay. I applied a truss, and heard nothing more from him until April 13th, 1858; when he called upon me, saying he thought he had another hernia. Upon examination, I found what, at the time, I thought to be a femoral hernia; and it seemed to have every indication of being one. It was the size of a small hen's egg, and could be easily returned into the cavity of the abdomen, attended with a somewhat dull sensation of gurgling to the sense of touch, but not to the sense of hearing. It had made its appearance gradually; the patient thought it nothing but some slight glandular enlargement; but, upon closer examination, found it could be returned, the same as the hernia of the opposite side. I was inclined to think it a femoral hernia, and applied a compress with the spica bandage; which seemed to retain it, and answered every purpose at that time.

On the 26th of June following, he again called upon me, saying, for a few weeks past he had been unable to make the bandage answer the purpose it had done, and the breach had increased in size very rapidly; but he had had so much business to do, he could not get time to visit me as soon as he wished. He had not suffered the least inconvenience or pain from it. I found the swelling considerably increased in size from when I

last saw it, and could not succeed entirely in returning it into the cavity of the abdomen; but the portion which I did not return still communicated to the touch the same gurgling sensation; it seemed as though I could detect a slight fluctuation, which indicated that it might be a cyst filled with fluid, which gave me some doubts as to my former diagnosis. But here again, the patient had been a farm-laborer,—had worked hard,—was in perfect health,—had never suffered from pains in the lumbar or pelvic regions,—had not the slightest indisposition, and had worked every day. I questioned him closely, and had him remove his clothing entirely. His muscles were firm and strong; his pulse was regular in frequency, force, and rhythm; there was no tenderness or soreness on pressure, movement, or percussion; but I fancied I could detect a peculiar, tense condition of the abdominal parieties of the right side, in front, especially in the right iliac region, which was unnatural; nevertheless, nothing could be detected indicating other than the slightest and almost imperceptible fluctuation in the tumor by percussion. His appetite was good; every function of the body was healthily performed; he worked hard and slept well. I again applied the compress and bandage, which only partially retained the tumor; and the use at night of cold lotions of acetate of lead; and directed him to lie upon his back, and cease all severe and violent exertions,—to remove the bandage at night, and reapply it in the morning.

On the 1st of August, he called upon me again. I found his general health good; the tumor was evidently increasing in size, and the sense of fluctuation which I had discovered previously, was evidently increased. I stated my apprehension to him, that I possibly might be mistaken relative to the nature of the tumor, which he denominated a breach; that it was not a hernia, as we had supposed, but a cyst, containing fluid; but what it was, and where it came from, unless it was a psoas abscess, I could not tell. For it to be an abscess, it did seem there ought at least to be some concomitant symptoms attending the accumulation of such an amount of pus. I plainly told him I felt that I was wrong in my previous diagnosis, and requested him to show it to some other physician or surgeon. I prescribed the lotion again and bandage for support of the tumor, until I saw it again.

August 18th, he called upon me, saying that for a few days past he had been unable to work, from the size of the swelling; he could not retain in position, and that it had enlarged very rapidly. Still he suffered no pain nor inconvenience, but from its size and the chafing from movement; he had no pain or fever; his appetite was good, and general health never was better.

On examining the tumor, I found it had rapidly increased in size—it being as large as a quart bowl. Several purple places were upon the surface, which were evidently points where openings would soon take place; and the evidence was unmistakable of its being pus. The patient residing some seven miles distant in the country, and having no other conveyance home than horseback, the way he came, I proposed to visit him at his own home, on the next day, and let out the contents of this enormous swelling. My friend Dr. X. Phillips, being somewhat interested in the case from my description, and being well acquainted with the patient, and knowing of his being a hard-laboring man, and not even his nearest neighbors knew that he was laboring under any such difficulty as he actually was,—the Dr. examined the case, and did not hesitate to pronounce that it was fluid contained in the sac. I made a large opening in the most dependent portion, with a large abscess lance; when it discharged in a continuous stream the size of the orifice, one quart and three oz. of thick, healthy-appearing pus. I closed the orifice or opening by a lint-compress, applied the spica bandage, recommended a light diet, perfect quietude, until I saw him again, on the following day. I then removed the dressing, and an ounce or more of pus flowed out. He was very impatient to get out, and felt as if he could go immediately to work. Still I advised him to keep quiet for several days to come at least,—dress it once per day, or more, if it seemed to need it.

On the fifth day after opening it, I saw him again. He still continued to do well, and was able to do some light work; and on the third week, the integuments had regained their normal texture and position, leaving nothing but a small opening, from which flowed a little yellowish serum; and he was able to work as well as ever. He returned in a few days afterwards to his native country, England, since which I have not heard from him.

The peculiarities of this case seem to be, the entire absence of all premonitory symptoms,—no fever—no indisposition in the least, only from the size during the last two weeks previous to the opening of it. His previous health had always been good,—had never been sick any, or received any injuries of whatever kind. The obscurity with which it first made its appearance; the facility with which it could be returned into the cavity of the abdomen, gave every appearance of its being a femoral hernia; notwithstanding, the result showed differently.

